# A test of financial incentives to improve warfarin adherence

**DOI:** 10.1186/1472-6963-8-272

**Published:** 2008-12-23

**Authors:** Kevin G Volpp, George Loewenstein, Andrea B Troxel, Jalpa Doshi, Maureen Price, Mitchell Laskin, Stephen E Kimmel

**Affiliations:** 1 Center for Health Equity Research & Promotion, Philadelphia Veterans Affairs Medical Center, Philadelphia, PA, USA; 2 Center for Health Incentives, Leonard Davis Institute of Health Economics, Philadelphia, PA, USA; 3 Department of Medicine, University of Pennsylvania School of Medicine, Philadelphia, PA, USA; 4 Department of Health Care Management, the Wharton School, University of Pennsylvania, Philadelphia, PA, USA; 5 Department of Social and Decision Sciences, Carnegie Mellon University, Pittsburgh, PA, USA; 6 Center for Clinical Epidemiology and Biostatistics and Department of Biostatistics and Epidemiology, University of Pennsylvania, Philadelphia, PA, USA; 7 Department of Pharmacy Service, Hospital of the University of Pennsylvania, Philadelphia, PA, USA

## Abstract

**Background:**

Sub-optimal adherence to warfarin places millions of patients at risk for stroke and bleeding complications each year. Novel methods are needed to improve adherence for warfarin. We conducted two pilot studies to determine whether a lottery-based daily financial incentive is feasible and improves warfarin adherence and anticoagulation control.

**Methods:**

Volunteers from the University of Pennsylvania Anticoagulation Management Center who had taken warfarin for at least 3 months participated in either a pilot study with a lottery with a daily expected value of $5 (N = 10) or a daily expected value of $3 (N = 10). All subjects received use of an Informedix Med-eMonitor™ System with a daily reminder feature. If subjects opened up their pill compartments appropriately, they were entered into a daily lottery with a 1 in 5 chance of winning $10 and a 1 in 100 chance of winning $100 (pilot 1) or a 1 in 10 chance of winning $10 and a 1 in 100 chance of winning $100 (pilot 2). The primary study outcome was proportion of incorrect warfarin doses. The secondary outcome was proportion of INR measurements not within therapeutic range. Within-subject pre-post comparisons were done of INR measurements with comparisons with either historic means or within-subject comparisons of incorrect warfarin doses.

**Results:**

In the first pilot, the percent of out-of-range INRs decreased from 35.0% to 12.2% during the intervention, before increasing to 42% post-intervention. The mean proportion of incorrect pills taken during the intervention was 2.3% incorrect pills, compared with a historic mean of 22% incorrect pill taking in this clinic population. Among the five subjects who also had MEMS cap adherence data from warfarin use in our prior study, mean incorrect pill taking decreased from 26% pre-pilot to 2.8% in the pilot. In the second pilot, the time out of INR range decreased from 65.0% to 40.4%, with the proportion of mean incorrect pill taking dropping to 1.6%.

**Conclusion:**

A daily lottery-based financial incentive demonstrated the potential for significant improvements in missed doses of warfarin and time out of INR range. Further testing should be done of this approach to determine its effectiveness and potential application to both warfarin and other chronic medications.

## Background

Medical conditions known to increase the risk of thromboembolism (TE) affect millions of patients worldwide each year [1], with substantial associated morbidity and mortality [2-7]. Warfarin is recommended for the majority of these patients [8-12] and, when used properly, dramatically reduces the risk of embolic events [13].

However, despite its manifest benefits, poor control of anticoagulation levels is fairly common. Even in standard-of-care anticoagulation clinics devoted to monitoring patients on warfarin, [1] 32% to 68% of patient-time is spent out of the target therapeutic range, and poor adherence is a strong contributor [14-16]. One recent cohort study found that 40% of subjects missed 20% or more of their warfarin doses [17]. Inadequate regulation of anticoagulation levels reduces the drug's benefit, and can produce side-effects and create physician reluctance to prescribe warfarin in the first place [18,19]. Novel and scalable methods for improving adherence are needed to improve both the safety and effectiveness of warfarin.

Lotteries, which are extremely popular among Americans [20], are a potentially cost-effective way to deliver financial rewards to subjects and thereby improve adherence. More than 50% of adults residing in States with lotteries play at least once a year, spending a total of $48 billion ($166 per person).

We undertook two pilot studies that tested the feasibility and potential effectiveness of a novel approach to improving warfarin adherence and anticoagulation control that involves daily lottery incentives and makes use of a computerized pill-box that could enhance the scalability of the approach. This is the first test ever undertaken of a daily lottery to improve medication adherence and draws on a number of insights from the field of behavioral economics, including the importance of frequent feedback and incentives [21,22], the greater motivational power of lotteries over similarly valued certain payments [23], and the motivating force of anticipated regret [24].

## Methods

### Study population

Patients, 21 years or older, with ongoing care at the Hospital of the University of Pennsylvania Anticoagulation Management Center were invited to participate in the study, which was approved by the Institutional Review Board of the University of Pennsylvania. Ten patients who had been taking warfarin for at least 3 months participated in each of the two pilot studies. To participate, subjects had to provide written consent, have a home telephone line (to connect the monitor), and be capable of using the pill monitor.

### Study procedures

Each subject was provided with an Informedix Med-eMonitor™ System, which has a display screen and separate medication compartments. The device was programmed to communicate by telephone with a central database accessible by the study's administrator. Participants were enrolled in a daily lottery and followed for 3 months. Each subject was assigned a 2-digit number upon entry into the study, e.g., "27". In the first pilot, the expected value per day of the lottery was $5, which was comprised of a 2 in 5 chance at a $10 reward (e.g., either 2 or 7 is selected as the first or second digit) and 1 in 100 chance at $100 reward (e.g., '27' is selected). In the second pilot, conducted with a new group of subjects meeting the same enrollment criteria, the lottery had an expected daily value of $3, which was comprised of a 1 in 5 chance at a $10 reward (e.g., either 2 is selected as the first digit or 7 as the second digit) and 1 in 100 chance at $100 reward. Although patients were enrolled in the lottery each day that they were instructed to take a pill, they were only eligible to receive payment if the Med-eMonitor indicated they had opened the pill compartment and confirmed that they took their warfarin as prescribed. If a patient was told to not take warfarin on a particular day they would only be ineligible if they failed to comply by opening the compartment and taking a pill that day. Patients who were ineligible based on nonadherence who won the lottery were notified that they won and would have been paid, had they taken their medication. The Med-eMonitor was also programmed to provide a daily reminder chime, but no other reminder messages.

### Measurement of outcomes

The primary outcome measured was the proportion of out of range INRs, based on the subjects' prescribed INR range. The secondary outcome was patient adherence, calculated as "mean correct patient pill taking" based on the percentage of days in which each patient correctly opened the correct compartment and recorded pill taking.

### Statistical analysis

Analyses quantified each subject's adherence during the intervention, and compared that with either historical group controls or their own personal history, both as measured by Medication Event Monitoring System^® ^(MEMS) cap usage from a prior study [17]. We examined the proportion of INRs out of range during the intervention and compared these with the proportion of INRs out of range in the same patients for the 3 INRs immediately preceding and immediately following the intervention.

## Results

**Figure 1:**
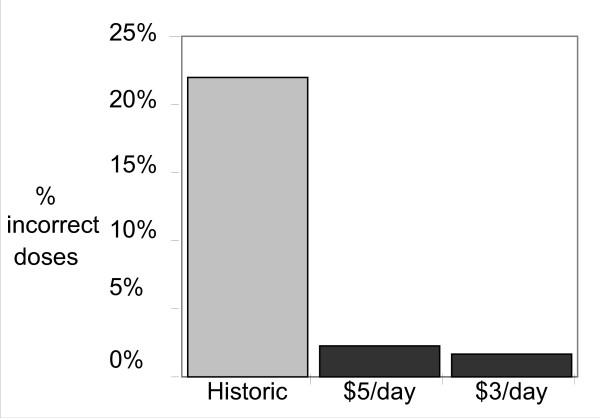
**Adherence under lotteries compared to historic controls**.

All patients were able to set-up and use the monitors successfully in their homes. The first pilot (expected value of lottery $5 per day) included 979 patient-days of warfarin use (mean 97.9, range 83–118). Over this time period, the mean proportion of incorrect pills taken was only 2.3% incorrect pills, compared with a historic mean of 22% incorrect pill taking in this clinic population (Figure 1). Mean adherence ranged from 92 to 100% (0–8% incorrect pills taken) per patient. Five of these patients also had MEMS cap adherence data from warfarin use in our prior study, and their mean incorrect pill taking decreased from 26% pre-pilot to 2.8% in the pilot. In the second pilot study (expected value of lottery $3 per day), an additional 10 patients contributed a total of 813 days of warfarin use (mean 81.3, range 14–103). Mean adherence was 98.4% (only 1.6% incorrect pills taken). Mean adherence ranged from 92.1% to 100%, similar to the $5/day pilot (Figure 1). Two of these patients had MEMS Cap adherence data from a prior warfarin study, one with 27.9% incorrect dosing and one with 6.4%.

**Figure 2:**
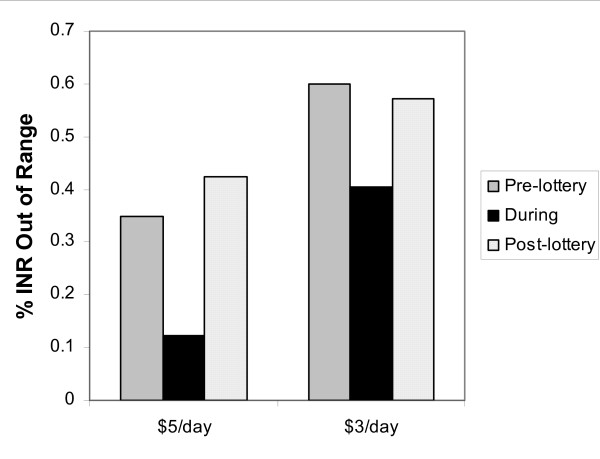
**Differences in time out-of-range INRs while in lottery compared to pre-enrollment**.

There was a substantial improvement in out of range INRs during both studies (Figure 2). In the first pilot the proportion of out of range INRs decreased from 35.0% pre-pilot to 12.2% post-pilot, a 65.2% improvement. In the post-intervention period, the proportion of INRs out of range increased back to close to the baseline value (Figure 2). In the second pilot, INRs out of range decreased from 65.0% to 40.4%, a 37.9% improvement, and again increased back to close to baseline post-intervention (Figure 2).

Only one of 20 patients developed a new elevation in INR on the first INR measurement after beginning pilot (INR was 3.3), suggesting that improvement in adherence among previously poorly adherent patients did not result in new over-anticoagulation. Four patients (1 in the first pilot and 3 in the 2^nd ^pilot) had an INR above target range at enrollment. In all four of these patients, their INR was within range by the time of the 2^nd ^follow-up visit. There were no serious adverse events among any of the participants.

## Discussion

In two small-scale studies, we demonstrate that a lottery-based financial incentive coupled with a simple reminder system substantially improved the rate of non-adherence to warfarin compared with historic controls, accompanied by a large improvement in anticoagulation control relative to their baseline values at a state-of-the-art anticoagulation clinic, While there is controversy about whether such payments to patients should be used [25,26], the degree of improvement observed in non-adherence rates in this pilot is striking.

The fact that subjects' proportion of INRs out of range returned to close to their baseline values post-intervention indicates that while the lottery-based incentive appeared to be effective in improving anticoagulation control, longer-term administration of the incentive program is likely necessary. It would be important to know whether a sustained effect can be attained through longer-term administration of the intervention; for example, would people internalize improved medication taking habits if the intervention were longer-standing? It is also possible that longer-range administration of the adherence-improving intervention may be necessary to sustained improved adherence. Longer-term administration of this intervention could be cost effective for high-risk patients on warfarin given the high risk of stroke and other thrombotic complications.

Lotteries used as incentives have had some success in altering health behaviors [27,28]. Small payouts, e.g., $10 to quit smoking, may not be effective [28], but knowledge about the effectiveness of lotteries in this regard is limited, as nearly all lottery-based studies to date have used rewards with low expected values and did not provide daily payouts. In the only study to have previously used daily lotteries, our group found that a lottery similar in design to the lottery used in this study with an expected value of $3 per day led to significant amounts of weight loss relative to a control group[29] Lotteries with larger expected values and daily lotteries, to our knowledge, have never been tested in the context of medication adherence.

The lottery incentive was designed to take advantage of several effects identified in the behavioral economics literature on incentives. First, consistent with research showing that even small rewards and punishments can have great incentive value if they occur immediately [21,22,30,31], adherent patients received rapid feedback about whether they won and non-adherent subjects received feedback about whether they would have won had they been adherent. Second, based on research showing that people are motivated by the experience of past rewards and the prospect of future rewards [32] and that people are particularly emotionally attracted to small probabilities of large rewards [33], the lottery was tailored to provide frequent small payoffs (a 1 or 2 in 5 chance at a $10 reward) and infrequent large payoffs (a 1 in 100 chance at a $100 reward). Third, research on decision making has found that the desire to avoid regret is a potent force in decision making under risk [23,34]. By giving non-adherent patients feedback about what they would have won had they been adherent, the incentive scheme was designed to maximize the threat of regret if people failed to adhere. Lotteries may be more effective than fixed payments (e.g. $3 per day), as people tend to overweigh small probabilities in making decisions [23,35] and playing a daily lottery may have entertainment value that offsets some of the tedium of taking daily medications.

The major limitation of this study is that it was not conducted as a randomized controlled trial. However, the within-subject improvement in INRs as well as the improvements in adherence both among subjects in whom we had MEMS cap adherence data pre-intervention and compared to historical controls were quite large. Nonetheless, demonstration of the effectiveness of this intervention will ultimately require a randomized trial ideally with longer-term follow-up to examine the sustainability of this approach. Examination of cost effectiveness will also be important to determine the likelihood of adoption by payers.

The success of the intervention raises ethical issues. First, there could be an objection to paying people to 'do things that they should do anyway.' However, the behavioral economics literature finds that even highly motivated individuals often have difficulty in making decisions in the short term that favor their long-term interests [36]. A lottery (or other reward system that provides frequent positive reinforcement) can be thought of as a way to help patients to internalize these long-term benefits so they make decisions in the short-term that favor their long-term interests. From the standpoint of a payor, a similar amount of money could be used to treat strokes that result from non-use of warfarin, or to provide an incentive system like this, which reduces the rate of strokes, which is clearly a better outcome for the patients. Second there could be a concern that rewarding patients to take medication could reduce their sense of personal responsibility for their health and hence adherence if and when incentives are removed. However, our study provided no evidence of such a negative rebound effect, and it may be true that any long-term changes in behavior induced by incentives would persist due to the establishment of adherent habits.

## Conclusion

These studies provide initial evidence of the feasibility and potential promise of a lottery-based financial incentive in improving medication adherence for patients using warfarin. Given the prevalence of conditions necessitating warfarin use, high rates of non-adherence, and attendant consequences for patient morbidity and mortality, this approach shows great promise and merits further testing. This novel approach could potentially also be utilized to improve medication adherence for a wide range of other chronic conditions that require ongoing use of medications.

## Competing interests

Dr. Kimmel has received funding and served as a consultant for several pharmaceutical companies, none related to the nature of this study. Dr. Volpp and Dr. Kimmel currently receive funding for investigator-initiated incentive-based research from Pfizer, Inc. and the Aetna Foundation, and Dr. Volpp has received funding from Astra Zeneca to convene a conference on medication adherence. There are no known financial conflicts of interest among any of the authors including but not limited to employment/affiliation, all grants or funding, honoraria, paid consultancies, expert testimony, stock ownership or options, and patents filed, received or pending.

## Non-financial competing interests

None

## Authors' contributions

KV helped conceive and design the study, was involved in drafting the manuscript, and revising it critically for important intellectual content, and has given final approval of the version to be published. GL helped conceive and design the study, was involved in drafting the manuscript, and revising it critically important intellectual content, and has given final approval of the version to be published. AT was involved in analysis and interpretation of data, drafting the manuscript, and revising it critically important intellectual content, and has given final approval of the version to be published. JD was involved in analysis and interpretation of data, drafting the manuscript, and revising it critically important intellectual content, and has given final approval of the version to be published. MP was involved in acquisition of data, drafting the manuscript, and has given final approval of the version to be published. ML was involved in acquisition of data, drafting the manuscript, and has given final approval of the version to be published. SK helped conceive and design the study and did the primary analyses of the data, was involved in drafting the manuscript, and revising it critically for important intellectual content, and has given final approval of the version to be published

## Pre-publication history

The pre-publication history for this paper can be accessed here:


